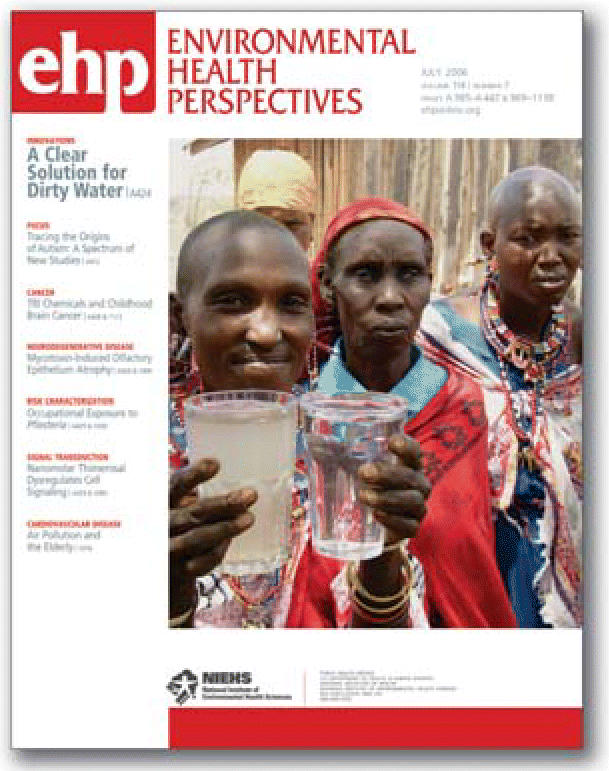# *EHP*: A Home at the NIEHS

**Published:** 2006-08

**Authors:** David A. Schwartz

**Affiliations:** Director, NIEHS and NTP, E-mail: david.schwartz@niehs.nih.gov

As readers of *EHP*, many of you have no doubt followed the recent discussions over the fate of this publication as a publicly supported journal. I initiated these discussions last year as part of the process of evaluating the priorities, resources, and direction of the NIEHS. Many factors have been considered during this process, including public comments from the scientific and broader environmental health community, input from the institute’s advisory boards, and evidence of *EHP* ’s standing in the world of scientific journals. After listening very carefully to these comments and considering all the potential possibilities, I have decided that the NIEHS should continue to support *EHP* because it is simply the right thing to do.

Over the past decade, *EHP* has become the leading environmental health science journal in the world, providing cutting-edge research and information on the effects of the environment on human health. As a steward of the NIEHS and, consequently, the field of environmental health sciences, I recognize that it is our responsibility to ensure that *EHP* continues to exercise complete editorial independence in publishing the very best research in our field, as well as to provide open access to these critical research findings to those around the world who need it. In weighing the options, it became clear that the only way to ensure these essential characteristics was to continue to support *EHP* at the NIEHS.

Just last month, *EHP* was ranked by the Institute for Scientific Information’s *Journal Citation Reports* ( *JCR* ) as the number one journal in the categories of environmental sciences and public, environmental, and occupational health. *JCR*, which evaluates over 8,400 scholarly and technical journals worldwide, bases its rankings on journals’ impact factor, a calculation of how often an “average” journal article is cited in a particular year and a measure of a journal’s relative importance. The increase in *EHP* ’s impact factor and its new rankings serve to illustrate both the stature of the journal and the importance of the field of environmental health sciences to informing many scientific disciplines. I believe we have an opportunity to further develop *EHP* as a top-tier journal and am initiating steps to strengthen the science published by the journal while ensuring its continued publication in the face of budgetary limitations.

First and foremost, I have begun the process of forming a search committee to recruit a new editor-in-chief to succeed the current leadership. Unlike previous editors-in-chief, who were NIEHS staff, this new position will reside outside the institute and will be a practicing scientist and thought leader in our field. This person, supported by a strong editorial board, will provide scientific leadership for the journal and be responsible for all of its editorial decisions. An advantage of this arrangement is that it will serve to further strengthen both the editorial independence of the journal and the quality of the published science. *EHP* has a long history of editorial independence and has recently emerged as a leader in crafting and enforcing strong policies to address competing financial interests and awareness of potential conflicts of interest. Both of these qualities are absolutely essential to enhancing the research process and ensuring public trust and support for scientific endeavors. While *EHP* ’s financial dependence on the NIEHS could be viewed by some as a potential conflict, we will continue to use the safeguards that exist as well as to implement new ones, including having an outside editor-in-chief, to successfully guard against this.

In addition to the changes to editorial leadership, I am taking steps to reduce the overall costs of *EHP* to the NIEHS. One way to do this is by reducing the size of certain sections of the journal including the Environews and sections focusing on NIEHS-specific information. Although the Environews section of the journal is incredibly successful, it is also relatively costly to produce. However, given the importance of this information, we will continue to publish a modest news section and selected editorials. NIEHS-specific sections will become available on our newly redesigned institute website, expected to launch in early 2007.

I recognize that the translation of science for our broader readership is important, and *EHP* will continue to work with strategic partners to facilitate the translation of our journal, but we will no longer assume financial responsibility for this activity. Cutting back in certain areas, moreover, will reduce the overall cost of the journal and allow us to continue to maintain *EHP* at the NIEHS. This will also enable us to expand sections focused on publication of novel scientific findings.

My final concern in considering *EHP* ’s future was the commitment of the journal—and the NIEHS—to open access. As a journal, *EHP* has been among the pioneers of open access to scientific information, allowing thousands of scientists, as well as governments, advocates, and others free access to the tools and information they need to improve the health of millions of people around the world. Many journals have followed *EHP* ’s lead and more would likely do so if not for the restraints of the bottom line. It has been *EHP* ’s unique relationship with the NIEHS that has allowed the journal to provide this invaluable access. Maintaining *EHP* at the NIEHS is the only way to ensure continuation of this policy. As a publicly funded agency, the NIEHS has a responsibility to use its appropriations wisely and for the good of all U.S. citizens. I believe that ensuring open access to the research published in *EHP* is firmly in line with the priorities and direction of this institute.

While I recognize that change is difficult, I believe that these changes will allow us to control the costs of publishing *EHP* while retaining its position as a scientific communications leader. I am confident that the plan we’ve put in place will allow *EHP* to flourish and further develop as an independent scientific journal providing state-of-the-science research and information to the broadest possible audience.

## Figures and Tables

**Figure:**